# Supplementary material for: Genome-wide identification and characterization of Fusarium circinatum-responsive lncRNAs in Pinus radiata
Source: BMC Genomics. 2022 Mar 10;23:194. doi: 10.1186/s12864-022-08408-9 (PMC8908662; doi:10.1186/s12864-022-08408-9)
Supplement: Supplementary file 16 — Additional file 16. [file 12864_2022_8408_MOESM16_ESM.doc]

**Supplementary figures**


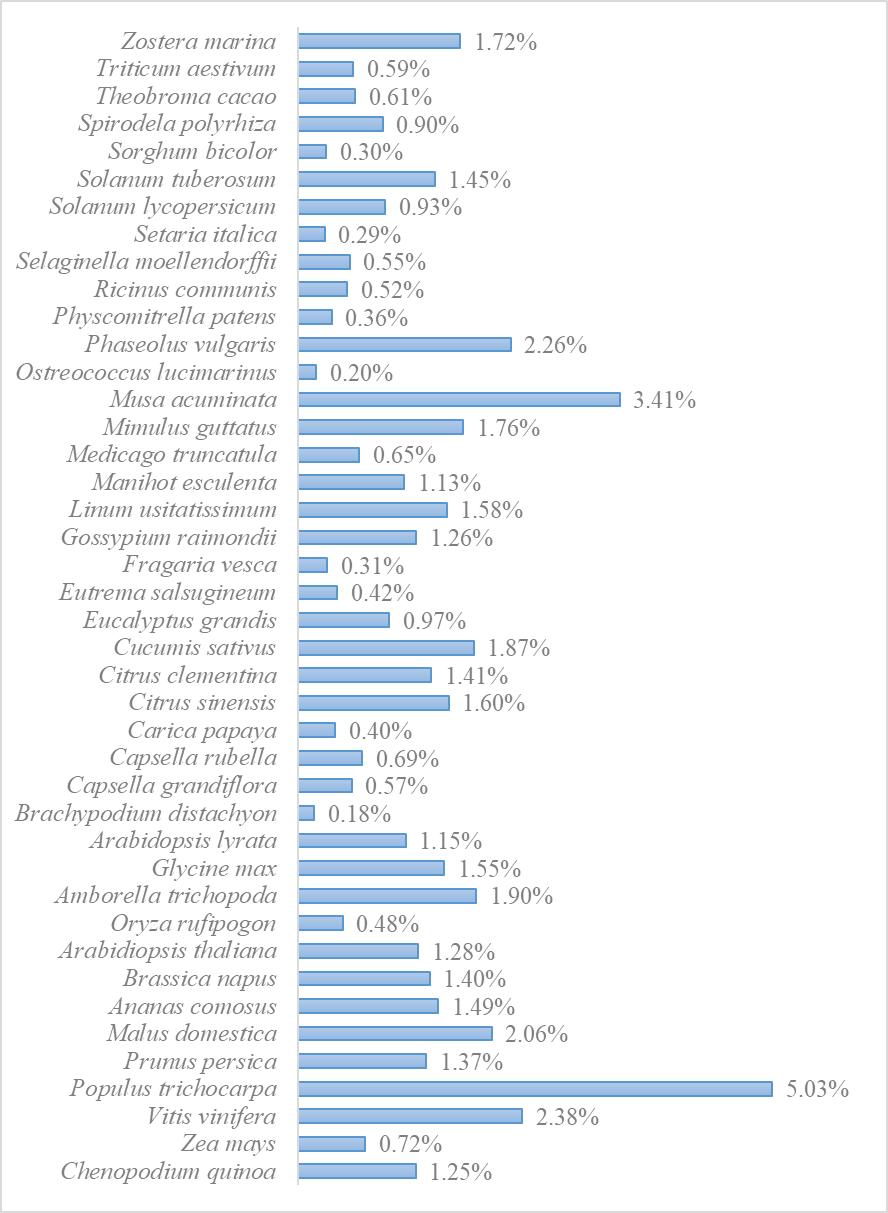


**Figure S1.** Homology ratio of *Pinus radiata* lncRNAs to lncRNAs of plant species from the CANTATA and GreeNc databases. The bars represent the number of hits of pine lncRNAs with those of each plant species to the total number of lncRNAs of each plant species.


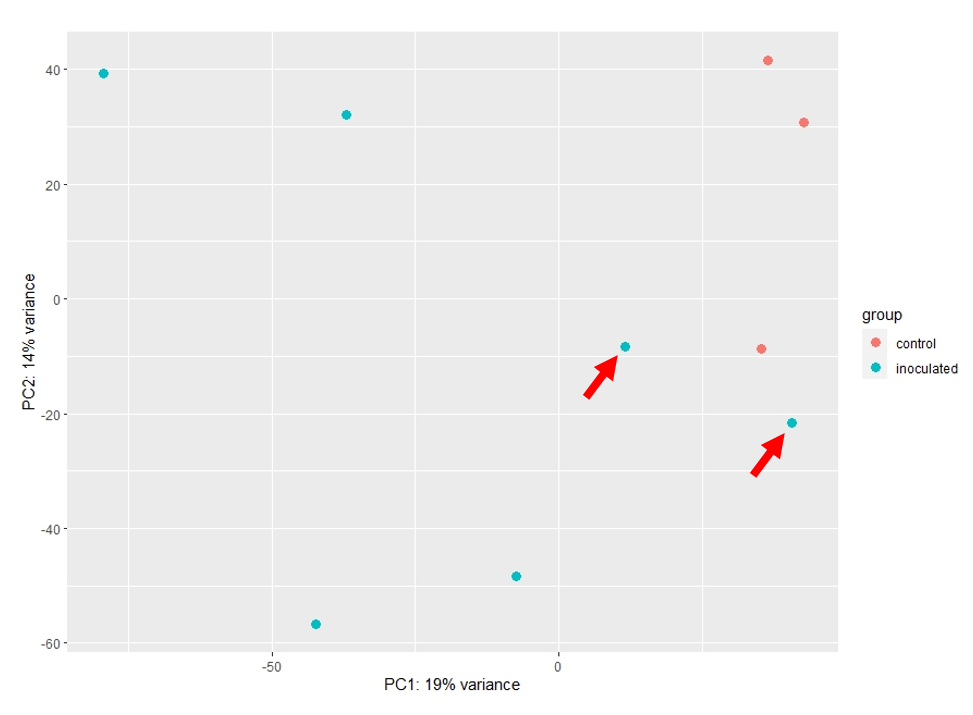


**Figure S2.** Two-dimensional scatterplot of the principal component analyses (PCA) for lncRNA expression data of *Pinus radiata*. Red arrows indicate the discarded samples for the downstream analysis.
